# CD56 Expression in Odontogenic Cysts and Tumors

**DOI:** 10.5681/joddd.2014.043

**Published:** 2014-12-03

**Authors:** Zohreh Jaafari-Ashkavandi, Ali Dehghani-Nazhvani, Faranak Razmjouyi

**Affiliations:** ^1^Associate Professor, Department of Oral and Maxillofacial Pathology, School of Dentistry, Shiraz University of Medical Sciences, Shiraz, Iran; ^2^Assistant Professor, Department of Oral and Maxillofacial Pathology, School of Dentistry, Shiraz University of Medical Sciences, Shiraz, Iran; ^3^Postgraduate Student, Department of Pediatric Dentistry, School of Dentistry, Shiraz University of Medical Sciences, Shiraz, Iran

**Keywords:** Ameloblastoma, CD56, immunohistochemistry, odontogenic cysts, odontogenic tumors

## Abstract

***Background and aims.*** Odontogenic cysts and tumors have a wide spectrum of clinical characteristics that lead to the different management strategies. Since definite diagnosis is difficult in some cases, it has been suggested that CD56 may be a candidate marker for definitive diagnosis of some odontogenic tumors. The present study was designed to examine CD56 expression in lesions with histopathological similarities.

***Materials and methods.*** In this cross-sectional, analytical study the subjects were 22 ameloblastomas, 13 dentigerous cysts, 10 keratocystic odontogenic tumors (KCOT), 4 adenomatoid odontogenic tumors (AOT), 3 orthokeratinized odonto-genic cysts, 3 calcifying odontogenic cysts (COC) and one glandular odontogenic cyst (GOC). All the samples were examined for CD56 immunoreactivity. Data were analyzed using chi-square test.

***Results.*** Twenty cases (91%) of ameloblastomas, 3 (75%) AOT, 4 (40%) KCOT and one case of GOC were positive for CD56. None of the dentigerous cysts, COC and orthokeratinized odontogenic cysts was CD56-positive. There was a significant difference in the CD56 expression between ameloblastoma and dentigerous cyst, as well as COC. Also, KCOT showed significantly higher expression than orthokeratinized odontogenic cyst.

***Conclusion.*** In this study CD56 expression was limited to the odontogenic tumors and more aggressive cystic lesions. This marker can be a useful aid for distinguishing cysts and tumors from similar lesions.

## Introduction

Odontogenic cysts and tumors can originate from various cell layers of tooth germ. These lesions represent a wide spectrum of clinical characteristics: benign, locally invasive or malignant.(1-8) On occasion, clinical and histopathological similarities between these cysts and tumors make the differential diagnosis difficult, particularly in a small biopsy specimen.(9-12) Management of these lesions is different, ranging from a conservative enucleation to a wide resection. Recently, immunohistochemistry (IHC) with the use of proper markers has become popular for the differentiation of these pathologic lesions. These markers can help pathologists distinguish lesions which have histopathological similarities from each other. Several markers have been studied in odontogenic lesions, such as calretinin that can be used to distinguish ameloblastoma from other odontogenic lesions.^4,11,(13-16)^ Also, it has been shown that cytokeratins (CK) are valuable markers for dif-ferentiating keratocystic odontogenic tumor (KCOT) from other cystic odontogenic lesions.^16,17^

CD56 or neural cell adhesion molecule (NCAM) is a transmembrane protein that belongs to the immunoglobulins and has a direct role in cell-to-cell adhesion. This marker is specific for natural killer (NK) cells.^1,(18-21)^ Also, its expression was found in various cell types, including skeletal muscle, osteoblasts, some CD4^+^ T-lymphocytes and pancreatic cells and also in some tumors like tumors of the nervous system, lung carcinomas, ovarian tumors and some types of leukemia.^21-23^ CD56 expression is essential for the growth and development of the nervous system.^1,21,22^ As tooth development is in close association with neural crest cells, several studies evaluated CD56 expression in the tooth germ.,^1,18,(24)^ The results showed its expression in epithelial and ectomesenchymal components of tooth germ. This protein was found in dental follicle and some parts of enamel organ in various stages of tooth development.^1,18,(24)^ Other studies evaluated this protein in odontogenic cysts and tumors. These studies demonstrated that CD56 was expressed in a small group of ameloblastomas and its expression in an odontogenic lesion is highly suggestive of this tumor.^11,22,(25)^

However, the results of these studies showed some discrepancies in CD56 expression in KCOT.^1,11^ Carins et al demonstrated that most cases of KCOT did not present CD56 expression, and Kusafuka et al showed immunoreaction with this marker in 50% of the cases. Also, the limited number of cases in these researches necessitates further studies to determine expression of the protein in various types of odontogenic lesions and to assess the value of this marker as a diagnostic aid. The main aim of this study was to evaluate CD56 expression in a group of common cystic and tumoral odontogenic lesions that pose difficulty in definitive diagnosis.

## Materials and Methods

In this cross-sectional and analytical study, fifty-six samples were investigated, including 22 cases of ameloblastoma (14 solid and 8 unicystic types), 13 dentigerous cysts, 10 KCOT, 4 adenomatoid odontogenic tumors (AOT), 3 orthokeratinized odontogenic cysts, 3 calcifying odontogenic cysts (COC) and one glandular odontogenic cyst (GOC). The subjects were retrieved from the archive of Oral and Maxillofacial Pathology Department of Shiraz Dental School from 1993 to 2011. The selected cases had adequate epithelial component and had not been decalcified. Severely inflamed lesions were excluded.

Baseline data including patients’ age and gender as well as the location of the lesions were recorded from the patients’ medical files.

### IHC staining

4-μm-thick sections of formalin-fixed and paraffin-embedded blocks were prepared for IHC staining, using Envision Labeled Peroxidase System (DAKO, Carpentaria, CA, USA). After de-paraffinization and rehydration, the sections were washed with distilled water and then, antigen retrieval was performed by DAKO cytomation target retrieval solution (DAKO, Carpentaria, CA, USA) at pH=9, for 20 minutes. Then, the sections were incubated with anti-CD56 antibody (ready to use, Clone 1B6, Novocastra, Newcastle, UK) for 30 minutes. 3, 3 di-aminobenzidine (DAB liquid, DAKO Corporation, Denmark) was used as chromogen. Osteoblasts were used as internal positive control.^(26,27)^ Primary antibody was replaced by PBS solution in negative control sections. Brown staining in the cell membrane, cytoplasm or both in the epithelial component was considered as positive. Positive staining was considered “extensive”, when more than 50% of epithelial cells showed immunoreaction, and was considered “focal”, when 1-50% of epithelial cells were positive. Data were analyzed with SPSS 11, using chi-square test. P-value (PV) was approximated using Monte-Carlo method and was considered significant at P<0.05. Study groups with less than 10 cases were not considered in the statistical analysis.

## Results

Clinical data of the patients are presented in Table 1. IHC examination showed brown membranous staining in 27 (49%) cases. Frequency and localization of the reaction were outlined in Table 2.

**Table 1:** Clinical data of patients in all study groups

Groups (n)	Age (mean)	Male:Female	Mandible:Maxilla
SA (14)	41.2	7:7	14:0
UA (8)	31.4	4:4	8:0
DC (13)	25.4	5:8	9:4
KCOT (10)	34	8:2	7:3
AOT (4)	45	1:1^*^	0:3^*^
GOC (1)	-^*^	-^*^	-^*^
COC (3)	21.5	0:2^*^	0:2^*^
OOC (3)	27.5	1:1^*^	1:1^*^
SA: solid ameloblastoma, UA: unicystic ameloblastoma, DC: dentiger ous cysts, KCOT: keratocystic odontogenic tumor, AOT: adenomatoid odontogenic tumor, GOC: glandular odontogenic cyst, COC: calcifying odontogenic cyst, OOC: orthokeratinized odontogenic cyst.			
^*^: some patients’ data were not available.			

**Table 2:** Comparison of CD56 immunoreactivity in the study groups

Groups	Number	CD56 positive (%)	P
SA	14	12 (85.7)	SA vs. UA: 0.51
UA	8	8 (100)	SA vs. DC: 0.001
DC	13	0 (0)	SA vs. KCOT: 0.61
KCOT	10	4 (40)	UA vs. DC: 0.001
AOT	4	3 (75)	UA vs. KCOT: 0.21
GOC	1	1 (100)	DC vs. KCOT: 0.001
COC	3	0 (0)	
OOC	3	0 (0)	
SA: solid ameloblastoma, UA: unicystic ameloblastoma, DC: dentigerous cysts, KCOT: keratocystic odontogenic tumor, AOT: adenomatoid odontogenic tumor, GOC: glandular odontogenic cyst, COC: calcifying odontogenic cyst, OOC: orthokeratinized odontogenic cyst.			

**Figure 1.**
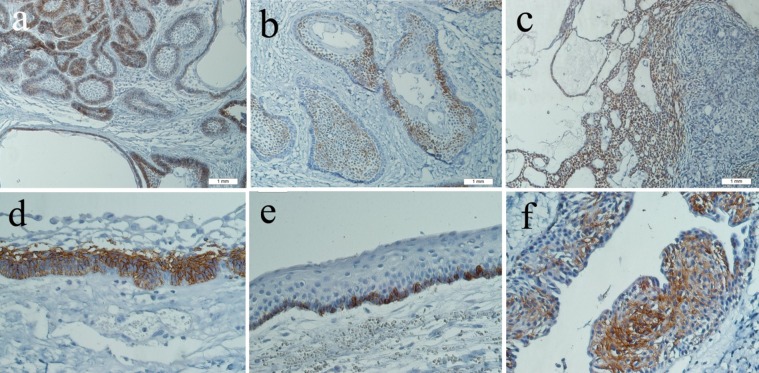
CD56 expression in solid ameloblastoma (a and b); a: CD56 expression limited to the peripheral ameloblast-like cells in ameloblastoma; b: the expression only in central SR-like cells. AOT (c): CD56 expression in hyperchromatic cells and anastomosing cords of AOT. d and e: CD56 expression is seen in basal cell layer of unicystic ameloblastoma (d) and KCOT (e). f: The expression in the epithelial lining of glandular odontogenic cyst, not in superficial cells (×200).

*Ameloblastomas:* Solid ameloblastoma consisted of 7 cases of follicular and 7 cases of plexiform subtypes. Immunoreactivity was limited to the cell membrane of the ameloblast-like cells in follicular type. All the cases in this group showed extensive staining (Figure 1-a). In plexiform ameloblastomas, staining was found in both peripheral and central stellate reticulum-like (SR) cells, and 2 cases showed extensive staining only in SR-like cells (Figure 1-b). Areas of squamous metaplasia and cystic formation did not show any reaction.

Unicystic ameloblastomas included 6 cases of mural and 2 luminal subtypes. Immunostaining was observed in both luminal epithelial lining and ameloblastic nests. The expression was mostly extensive (Figure 1-d).

*KCOT:* Four (40%) cases of KCOT revealed focal reaction in less than 30% of basal cells (Figure 1-e). One case displayed both membranous and cytoplasmic staining.

*AOT:* Three out of 4 cases showed extensive reaction in epithelial sheets and anastomosing cords of cubic cells, but not in whorled spindle epithelial cells (Figure 1-c). Ductal component did not show staining.

One case of GOC showed extensive membranous and cytoplasmic staining in the cells above the basal layer, but not in superficial columnar cells (Figure 1-f).

None of the cases of dentigerous cysts, COC and orthokeratinized odontogenic cysts were stained with CD56 antibody.

Chi-square test showed a significant difference between four groups (that had > 10 cases). The P-values are presented in Table 2. As it can be seen in Table 2 the differences in CD56 expression between dentigerous cysts with KCOT, and unicystic ameloblastoma and solid ameloblastoma were significant. However, KCOT and ameloblastomas as well as both types of ameloblastomas were similar in this regard.

## Discussion

CD56 is a protein associated with nervous system development. Because its expression has been reported in tooth germ and ameloblastoma,^1,11,18,22,24^ we hypothesized that it may be a useful marker in differential diagnosis of some odontogenic cysts and tumors. This study showed that CD56 mostly expressed in the cell membrane. It has also been shown that CD56 had different isoforms, with CD56^120KD^ usually expressed in cell membrane, but CD56^180KD^ and CD56^140KD^ contains a cytoplasmic domain, too.^25^ We used a CD56 pan-antibody in this research, which can label all the isoforms.^25^25 We can conclude that cytoplasmic isoforms were not present in most of the selected odontogenic lesions. Although one case of KCOT and GOC showed both membranous and cytoplasmic expression, that should be evaluated in another study with more samples, some studies have reported CD56^140KD^ expression in the more aggressive and malignant tumors,28^28-31^ and we know that these two cystic lesions have a high recurrence rate.

In this study we found CD56 expression in 91% of ameloblastomas in both solid and unicystic variants. Other studies demonstrated the same results.^22^ Er et al^22^reported its expression in all the frozen sections of solid ameloblastoma, and Cairns et al^11^in 97% of formalin-fixed specimens. Although they declared that decalcification and small size of specimens caused absence of staining, in our study one sample without decalcification also was not stained with this marker. This finding showed that although CD56 expression has been found in a high percentage of ameloblastomas, this reaction can be avoided and a few cases of ameloblastoma may be negative for CD56.

In the present study, immunoreaction was observed in peripheral ameloblast-like cells in follicular and most of the plexiform ameloblastomas. Other studies also presented this pattern.^1,24^ In addition, this finding is the same as CD56 expression in bud stage of tooth germ development, in which the expression was restricted to the inner enamel organ (IEE).^18^In most of the plexiform ameloblastomas, the marker was expressed in both peripheral and central (SR-like) cells. An interesting pattern was the protein expression in only SR-like cells and not in the peripheral cell layer. The absence of expression has been observed in IEE cells of the enamel organ in cap stage of rodent tooth development.^18^ These findings may indicate that ameloblastoma may originate from proliferating epithelial cells mimicking the enamel organ in the early stages of development. This pattern is similar to the calretinin expression in ameloblastoma.^15,16^ None of the former studies has described the CD56 staining in SR-like cells; this is probably due to the fact that they studied follicular ameloblastomas more than plexiform subtypes and most of them have evaluated the limited cases.

We found CD56^+^ cells in unicystic ameloblastoma in both luminal epithelial layer and infiltrating ameloblastic nests. However, none of dentigerous cysts expressed CD56 protein. Since the expression was found in all the mural types, absence of staining in luminal lining of some cases decreases the reliability of this marker in differential diagnosis of luminal unicystic ameloblastoma and dentigerous cyst with a similar histopathologic feature.

In the present study, AOT was positive for CD56, too. Although we evaluated a few number of cases, most of them extensively expressed the marker in cords of hyperchromatic cubic cells. Only Carins et al^11^11 have previously studied a few cases of AOT. They have evaluated ameloblastoma, and a few cases of ameloblastic fibroma, AOT and odontogenic fibroma. They reported that CD56 expression was found only in ameloblastoma and ameloblastic nests of other tumors, and no cases of AOT revealed immunoreaction. Origination of AOT was not proven, yet. Some studies have evaluated immunohistochemical expression of different markers to determine its origin, and dental lamina, enamel organ, stratum intermedium (SI) cells and reduced enamel epithelium (REE) have been concluded.^32^ CD56 expression in SI cells was not uniform and continuous in the enamel organ.^18^ Regarding the presence of CD56 in the enamel organ, and also the absence of its expression in dentigerous cysts that arise from REE, our results supported the enamel organ as an origin of AOT. Moreover, due to the small number of study subjects, this hypothesis about the origin of AOT needs further research.

Among the lesions with cystic configuration, only KCOT (in 40% of cases) and GOC showed focal expression of CD56. Previous studies reported conflicting results about the expression of CD56 in KCOT.^1,11^11 However, all the reported CD56^+^ cases showed focal staining in the basal layer. KCOT is a cystic lesion that originates from dental lamina remnants. It was formerly named odontogenic keratocyst (OKC); however, due to its high recurrence rate and aggressive nature, this lesion was reclassified as a cystic neoplasm in the latest World Health Organization (WHO) classification.^3^

Since CD56 has a role in cell proliferation and is associated with aggressive behavior of lesions,^31^ its expression in KCOT and GOC may be related to the infiltrating characteristics of these lesions. Other cystic lesions, including dentigerous cyst, orthokeratinized odontogenic cysts and COC, did not express the marker. All of them are benign lesions with low recurrence rate. Studies that have previously investigated dentigerous cysts and COC have reported the same results.^1,11^ CD56 expression has not been studied in the orthokeratinized odontogenic cysts previously. Regarding the histopathologic similarity between dentigerous cyst and unicystic ameloblastoma, COC and UA, as well as KCOT and orthokeratinized odontogenic cysts, we can use CD56 marker to distinguish them from each other. However, due to the limited number of COC, orthokeratinized odontogenic cysts and GOC cysts, further studies are necessary.

## Conclusion

In the present study, CD56 expression was limited to the more aggressive cysts and to the tumoral lesions. This marker, with consideration of clinical and histopathologic findings, can exhibit useful aid for distinguishing cysts and tumors from similar lesions.

## Aknowledgement

The authors thank the Vice Chancellery of Shiraz University of Medical Science for supporting this research (Grant #91-01-03-4776). This article is based on the thesis by Dr Faranak Razmjouyi. The authors also thank Dr M. Vossoughi of the Dental Research Development Center of the School of Dentistry for the statistical analysis and Dr S. Hamedani (DDS, MSc) for revision of the manuscript from an English language point of view.
